# IFI27 may predict and evaluate the severity of respiratory syncytial virus infection in preterm infants

**DOI:** 10.1186/s41065-020-00167-5

**Published:** 2021-01-02

**Authors:** Junyan Gao, Xueping Zhu, Mingfu Wu, Lijun Jiang, Fudong Wang, Shan He

**Affiliations:** 1 Department of Pediatrics, Affiliated Hospital of Yangzhou University, NO.368 Hanjiang Middle Road, Yangzhou, 225000 Jiangsu China; 2 Department of Neonatology, Children’s Hospital of Soochow University, NO.92 Zhongnan Street, Industrial Park, Suzhou, 215025 Jiangsu China; 3 Department of Pediatrics, The First People’s Hospital of Yunnan Province, NO.152 Jinbi Road, Kunming, 650031 Yunnan China

**Keywords:** Respiratory syncytial virus, Preterm infants, Differentially expressed genes, Protein-protein interaction, Pathway enrichment, Interferon alpha inducible protein 27

## Abstract

**Background:**

Preterm infants are a special population that vulnerable to respiratory syncytial virus (RSV) infection and the lower respiratory tract infections (LRTIs) caused by RSV could be severe and even life-threating. The purpose of the present study was to identify candidate genes of preterm infants who are susceptible to RSV infection and provide a new insight into the pathogenesis of RSV infection.

**Methods:**

Three datasets (GSE77087, GSE69606 and GSE41374) containing 183 blood samples of RSV infected patients and 33 blood samples of healthy controls from Gene Expression Omnibus (GEO) database were downloaded and the differentially expressed genes (DEGs) were screened out. The function and pathway enrichments were analyzed through Database for Annotation, Visualization and Integrated Discovery (DAVID) website. The protein-protein interaction (PPI) network for DEGs was constructed through Search Tool for the Retrieval of Interacting Genes (STRING). The module analysis was performed by Cytoscape software and hub genes were identified. Clinical verification was employed to verify the expression level of top five hub genes among 72 infants including 50 RSV infected patients and 22 non-RSV-infected patients hospitalized in our center. Further, the RSV infected infants with high-expression IFI27 and those with low-expression IFI27 were compared (defined as higher or lower than the median mRNA level). Finally, the gene set enrichment analysis (GSEA) focusing on IFI27 was carried out.

**Results:**

Totally, 4028 DEGs were screened out and among which, 131 most significant DEGs were selected. Subsequently, 13 hub genes were identified, and function and pathway enrichments of hub genes mainly were: response to virus, defense response to virus, regulation of viral genome replication and regulation of viral life cycle. Furthermore, IFI27 was confirmed to be the most significantly expressed in clinical verification. Gene sets associated with calcium signaling pathway, arachidonic acid metabolism, extracellular matrix receptor interaction and so on were significantly enriched when IFI27 was highly expressed. Moreover, high-expression IFI27 was associated with more severe cases (*p* = 0.041), more requirements of mechanical ventilation (*p* = 0.034), more frequent hospitalization (*p* < 0.001) and longer cumulative hospital stay (*p* = 0.012).

**Conclusion:**

IFI27 might serve to predict RSV infection and evaluate the severity of RSV infection in preterm infants.

**Supplementary Information:**

The online version contains supplementary material available at 10.1186/s41065-020-00167-5.

## Background

Respiratory syncytial virus (RSV) remains a major cause of neonatal lower respiratory tract infections (LRTIs) and is also a leading cause of hospital visits within the first year of life globally [1, 2]. Studies revealed that neonates especially preterm infants with gestational age (GA) < 32 weeks are genetically predisposed to developing severe RSV related LRTIs [3–5]. So far, there is still no specific treatment dealing with RSV infection and LRTIs caused by RSV could lead to high incidence of mechanical ventilation (MV) as well as high morbidity and mortality [6, 7]. In recent years, the preventive strategy of immunoprophylaxis by using monoclonal antibodies of RSV (such as palivizumab and motavizumab) has shown some certain efficacy [8, 9]. Early intervention may benefit the specifically susceptible population and identifying the candidate genes susceptible to RSV infection could provide suggestions for clinical decision and population surveillance.

With the development of microarray technology and bioinformatical analysis, the differentially expressed genes (DEGs) and functional pathways being involved in the pathogenesis and progression of many diseases could be identified. The aim of this study was to screen and identify the potential candidate genes as biomarkers for early prediction and evaluation of severe RSV infection in preterm infants. To avoid the false positive rates resulting from independent microarray analyses, three datasets from Gene Expression Omnibus (GEO) database were merged and adjusted as one matrix. Then, the DEGs between RSV patients and healthy controls were obtained. Gene Ontology (GO), Kyoto Encyclopedia of Genes and Genomes (KEGG) pathway enrichment and protein-protein interaction (PPI) network analyses were performed to search the hub genes associated with the pathogenesis of RSV infection. A total of 131 most significant DEGs were screened out and 13 hub genes were identified as potential biomarkers for RSV infection. Interferon alpha inducible protein 27 (IFI27) was demonstrated as the most meaningful gene either in bioinformatical analysis or in clinical verification. In addition, high-expression IFI27 was found to be associated with severe RSV infection, more mechanical ventilation, more frequent hospitalization and longer hospital stay of preterm infants.

Our study may provide a new insight into the understanding of RSV infection pathogenesis and the hub genes identified in present study may serve as potential biomarkers of RSV infection for preterm infants.

## Materials and methods

### Microarray data

Three gene expression datasets (GSE77087 [10], GSE69606 [11] and GSE41374 [12]) containing 183 blood samples of RSV infected patients and 33 blood samples of healthy controls were downloaded from GEO database (http://www.ncbi.nlm.nih.gov/geo) (Illumina GPL10558 platform, Illumina HumanHT-12 V4.0 expression beadchip, Affymetrix GPL570 platform, Affymetrix Human Genome U133 Plus 2.0 Array). The probes were converted into the corresponding gene symbols based on the annotation information of the platforms.

### Identification of DEGs

Three datasets were normalized by log2 conversion and merged as one expression matrix. The study batch effect was adjusted by using Cochran’s Q test for the expression matrix and the meta-analysis (Combining Effect Sizes method) was adopted to determine the DEGs between groups (*p* < 0.05 was considered statistically significant). Probe sets without corresponding gene symbols were removed and genes with more than one probe set were averaged respectively by using R software (version 3.6.2). The most significant DEGs were further selected by criterion of |logFC (fold-change)| > 1 and adj. *P*-value < 0.01.

### KEGG and GO enrichment analyses of DEGs

The Database for Annotation, Visualization and Integrated Discovery (DAVID; http://david.ncifcrf.gov, version 6.8) was employed to analyze the biological information of the most significant DEGs mentioned above. Both of KEGG and GO functions are integrated in DAVID [13]. KEGG is a database resource for understanding high-level functions and biological systems from large-scale molecular datasets generated by high-throughput experimental technologies [14]. GO is a major bioinformatical tool to annotate genes and analyze biological process of these genes [15]. *P* < 0.05 was considered statistically significant.

### PPI network construction and module analysis

The Search Tool for the Retrieval of Interacting Genes (STRING; http://string-db.org, version 11.0) was used to predict and establish the PPI network. An interaction with a combined score > 0.4 was considered statistically significant while nodes without connections were ruled out. The Cytoscape software (version 3.7.2) was employed for visualizing molecular interaction networks and the plug-in Molecular Complex Detection (MCODE) of Cytoscape was adopted for identifying the most significant modules in the PPI network based on topology. The criteria of selection were as follows: MCODE score ≥ 10, degree cut-off =2, node score cut-off =0.2, Max depth = 100 and k-score = 2.

### Hub genes selection and analysis

After the most significant modules were determined by MCODE, the plug-in cytoHubba of Cytoscape was used to rank the genes by EPC method and genes with MCODE score ≥ 10 were determined as hub genes. Then, the hub genes were also analyzed through DAVID website and their unique functions were documented.

### Patients and ethics statement

A total of 72 infants (≤12 months) hospitalized in Children’s Hospital of Soochow University were enrolled in this study and were divided into RSV infection group (*n* = 50) and control group (non-RSV-infection, *n* = 22). RSV infection was diagnosed by respiratory symptoms (such as wheezing, cough and tachypnea) accompanied with the positive of RSV specific polymerase chain reaction (PCR) based assay [16, 17]. The inclusion criteria for the RSV infection group were as follows (1): born at GA < 37 weeks (2); hospitalization due to RSV infection in the first year of life (3); no major congenital anomalies (4); no complex cardiac anomalies (5); no chromosomal abnormalities (6); no major immune deficiency. The inclusion criteria for the control group were as follows [1]: born at GA < 37 weeks [2]; hospitalization due to various diseases but excepted RSV infection (jaundice, diarrhea for instance); (3)–(6) were the same as listed in criteria for RSV infection group. This study was approved by the hospital ethics committee and written informed consents were obtained from the parents or guardians of all patients. All patients’ general features are summarized in Supplementary Table 1.

### Quantitative real-time polymerase chain reaction (qRT-PCR)

The blood samples were collected and the peripheral blood mononuclear cells (PBMCs) were isolated through Ficoll gradient centrifugation immediately and stored at − 80 °C. Total RNA from PBMCs were extracted using TRIzol reagent (Invitrogen, USA). For mRNA detection, each RNA sample was reverse transcribed into cDNAs using the reverse transcription kit (Takara, Japan). The qRT-PCR was employed to measure the levels of mRNA using the comparative Ct method. GAPDH was considered as the normalization control. All the primers for qRT-PCR were listed in Supplementary Table 2.

### Clinical data collection

The 50 RSV infected infants were further divided into two subgroups (low-expression group and high-expression group) by the median mRNA level of IFI27. Four categories of patients’ clinical information were reviewed and compared [1]: GA, birth weight (BW) and gender [2]; age and body weight at first admission due to RSV infection [3]; respiratory support including non-invasive ventilation and MV [4]; the frequency of hospitalization and the cumulative length of hospital stay due to RSV infection. Severe RSV infection of those in-patients was defined as refractory hypoxemia that no response to the oxygen therapy, significant acidosis (an arterial pH < 7.2), and MV required.

### Gene set enrichment analysis (GSEA)

GSEA is a computational method that assesses whether a set of prior defined genes showed statistically significant and concordant differences between two biological states [18]. GSEA was performed by GSEA software (Version 4.0.3) to further explore the function enrichment of gene sets under the condition of high-expression IFI27 (defined as the mRNA level of IFI27 higher than the median level). False discovery rate (FDR) < 25% and nominal *p* < 0.05 were set as the cut-off criteria.

### Statistical analysis

The continuous variables with normal distribution were expressed as mean and standard deviation while variables with skewed distribution were expressed as median and range, respectively. The categorical variable was described as number and percentage. The independent-samples T test and Mann-Whitney U test were used to assess normal distributional and skewed distributional variables, respectively. The categorical variables were analyzed using Chi square or Fisher’s Exact Test, as appropriate. SPSS 26.0 software was employed for data processing. GraphPad Prism 8.0.2 software was served as the tool for results visualization. *P* < 0.05 was considered to be statistically significant.

## Results

### DEGs identification

**Fig. 1:**
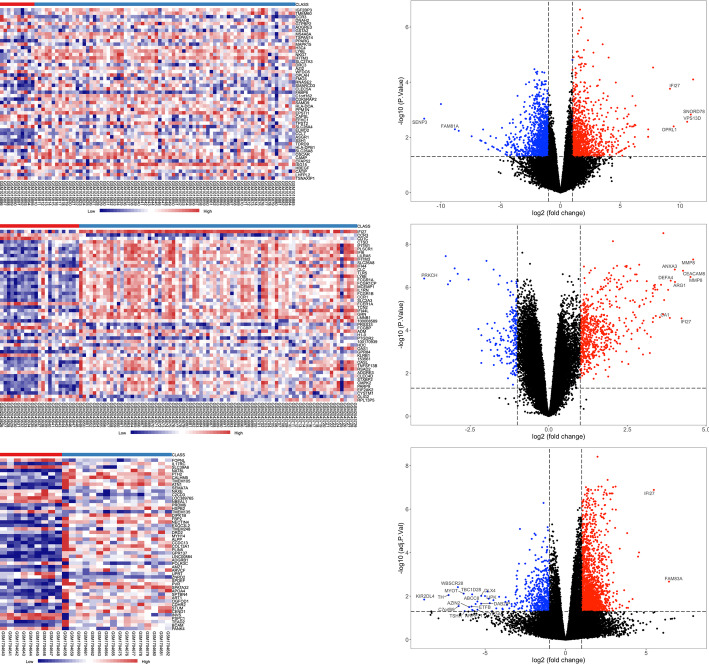
The heatmaps and volcano plots of the three datasets. In the heatmaps, the horizontal axis represented the sample gene in each sample. Red block represented the up-regulated gene while blue block represented the down-regulated gene. In the volcano plots, the horizontal axis represented the log (fold-change) of the DEGs. Red dot represented the up-regulated gene while blue dot represented the down-regulated gene. The DEGs with |log (fold-change)| > 5.5 were labeled

**Fig. 2:**
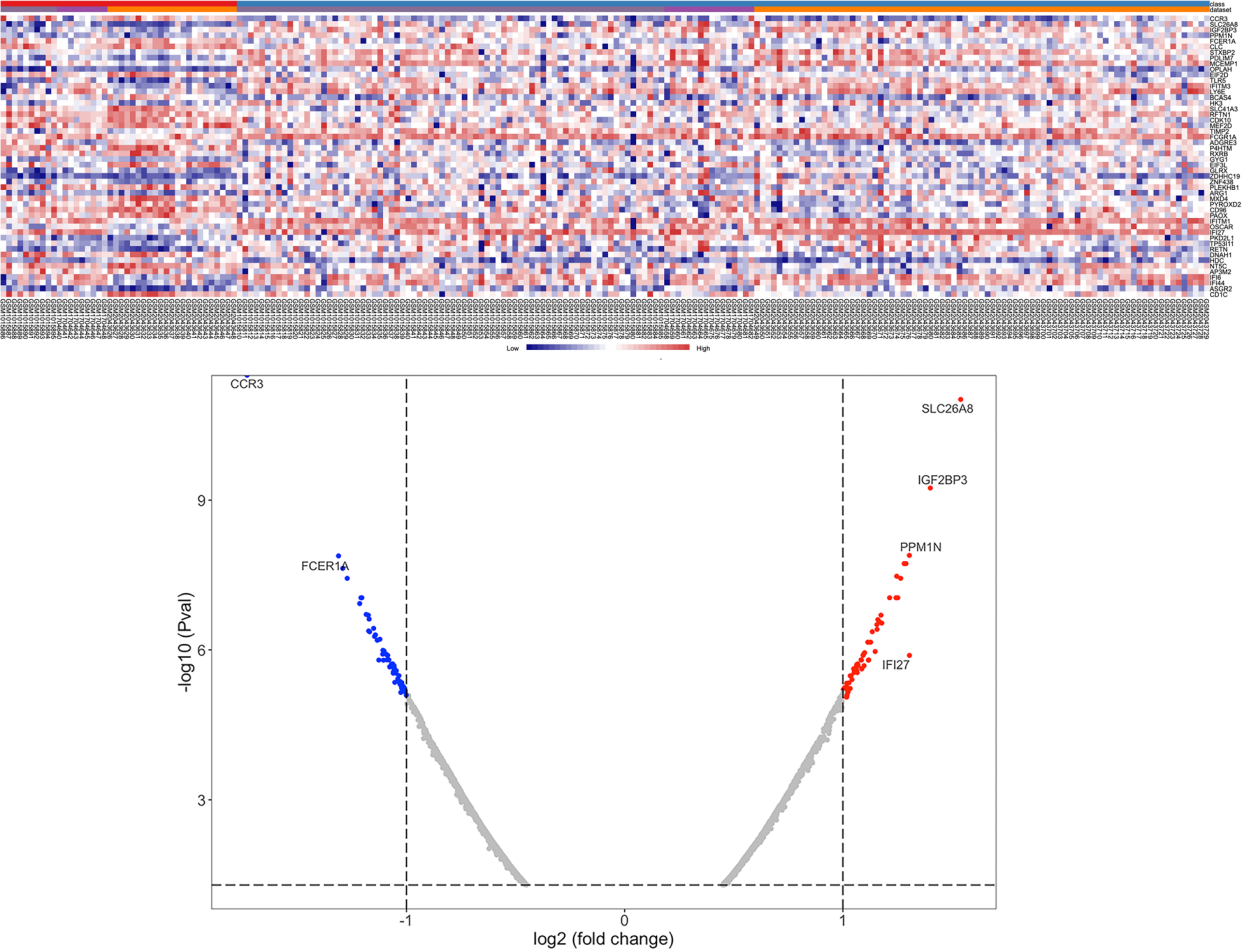
The heatmap and volcano plot of the merged and adjusted gene expression matrix. Red represented the up-regulated gene while blue represented the down-regulated gene. The DEGs with |log (fold-change)| > 1.3 were labeled

The DEGs of each dataset were shown as heatmaps and volcano plots and through which we can see that IFI27 was significantly upregulated in each dataset (Fig.1). Then, the three gene expression datasets were normalized, and the study batch effect was adjusted (Supplementary Fig. S1). After the three datasets merged as one expression matrix, the DEGs were identified (Fig. 2). Totally, 4028 DEGs including 2312 down-regulated genes and 1716 up-regulated genes were screened out by meta-analysis with *p* < 0.05. Then, 131 most significant DEGs consisting of 98 down-regulated genes and 33 up-regulated genes were determined by criteria of |logFC (fold-change)| > 1 and adj. *P*-value < 0.01.

### KEGG and GO enrichment analyses of DEGs

**Fig. 3:**
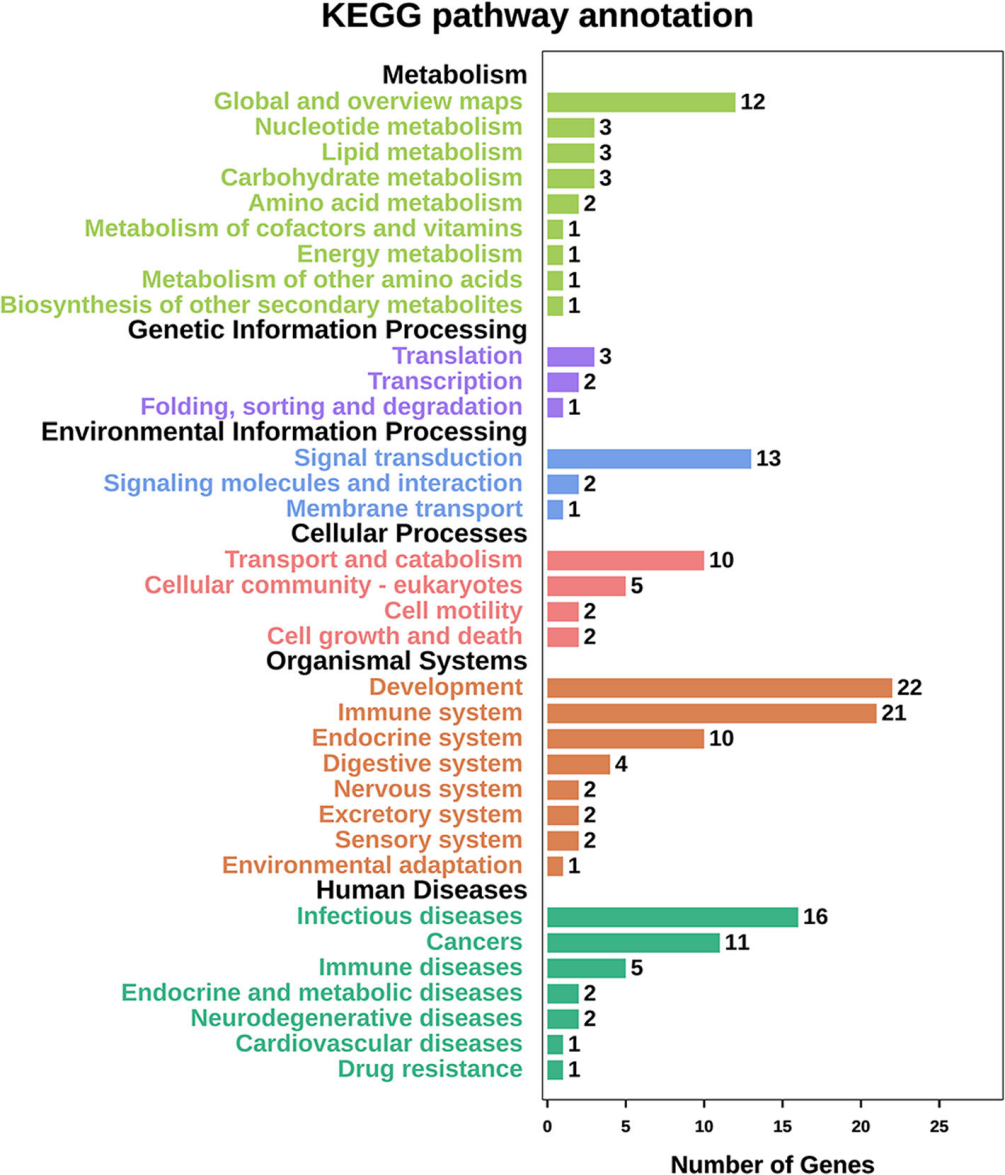
The KEGG pathway enrichment analyses. The length of bar represented the numbers of the DEGs enriched in each pathway and different color represented different functional enrichment

**Fig. 4:**
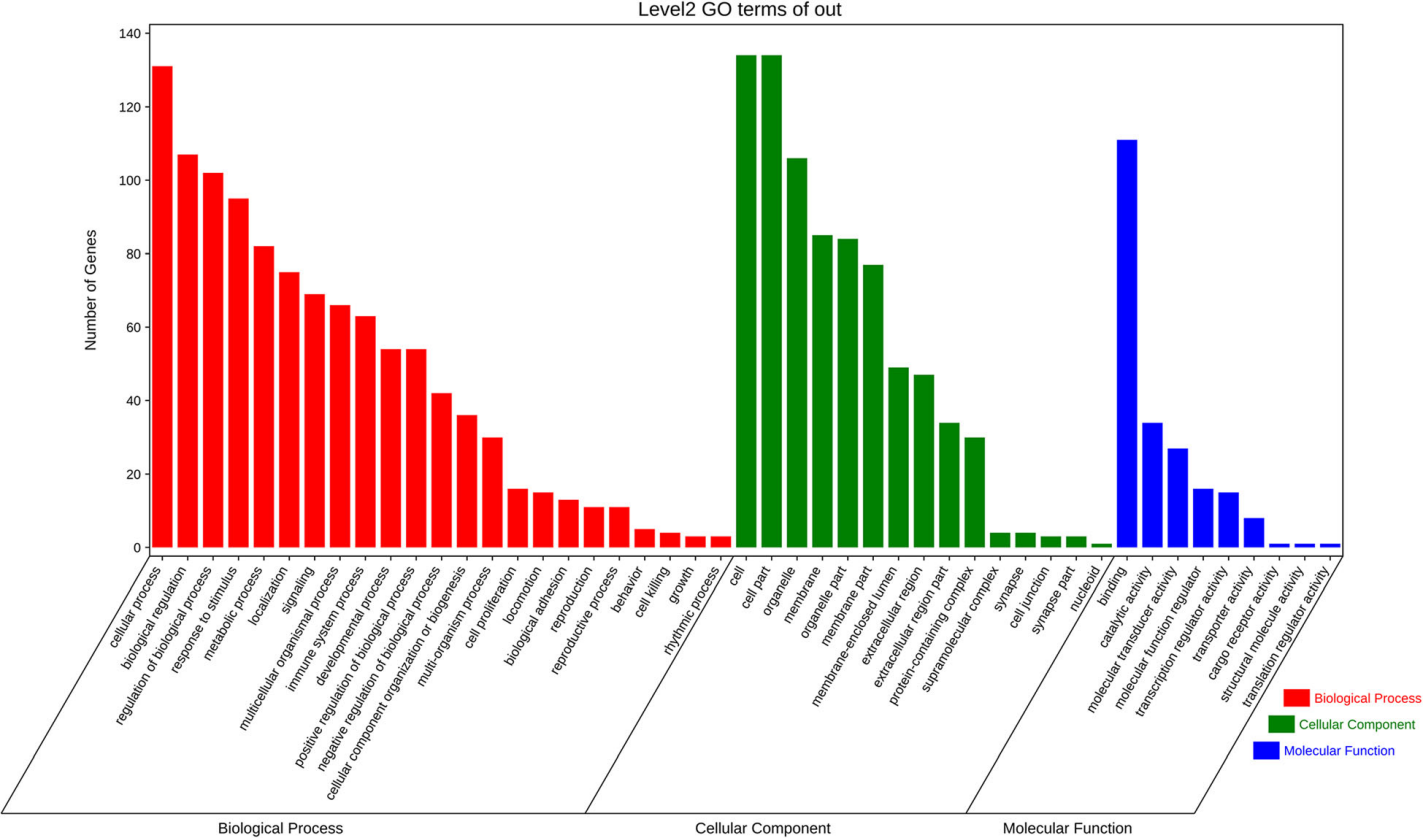
The GO function enrichment analyses. The length of bar represented the numbers of the DEGs enriched in each pathway and different color represented different functional enrichment

Through DAVID, function and pathway enrichments of the 131 most significant DEGs were analyzed. KEGG showed that those DEGs were mainly enriched in organ development, immune system, infectious diseases and signal transduction (Fig. 3). The biological processes (BP) of the DEGs by GO analysis were mainly enriched in cellular process, biological regulation and response to stimulus. The cell components (CC) were mainly enriched in cell, cell part, organelle and membrane. The molecular functions (MF) were mainly enriched in binding, catalytic activity, molecular transducer activity and molecular function regulator (Fig. 4).

### PPI network construction and hub genes identification

**Fig. 5:**
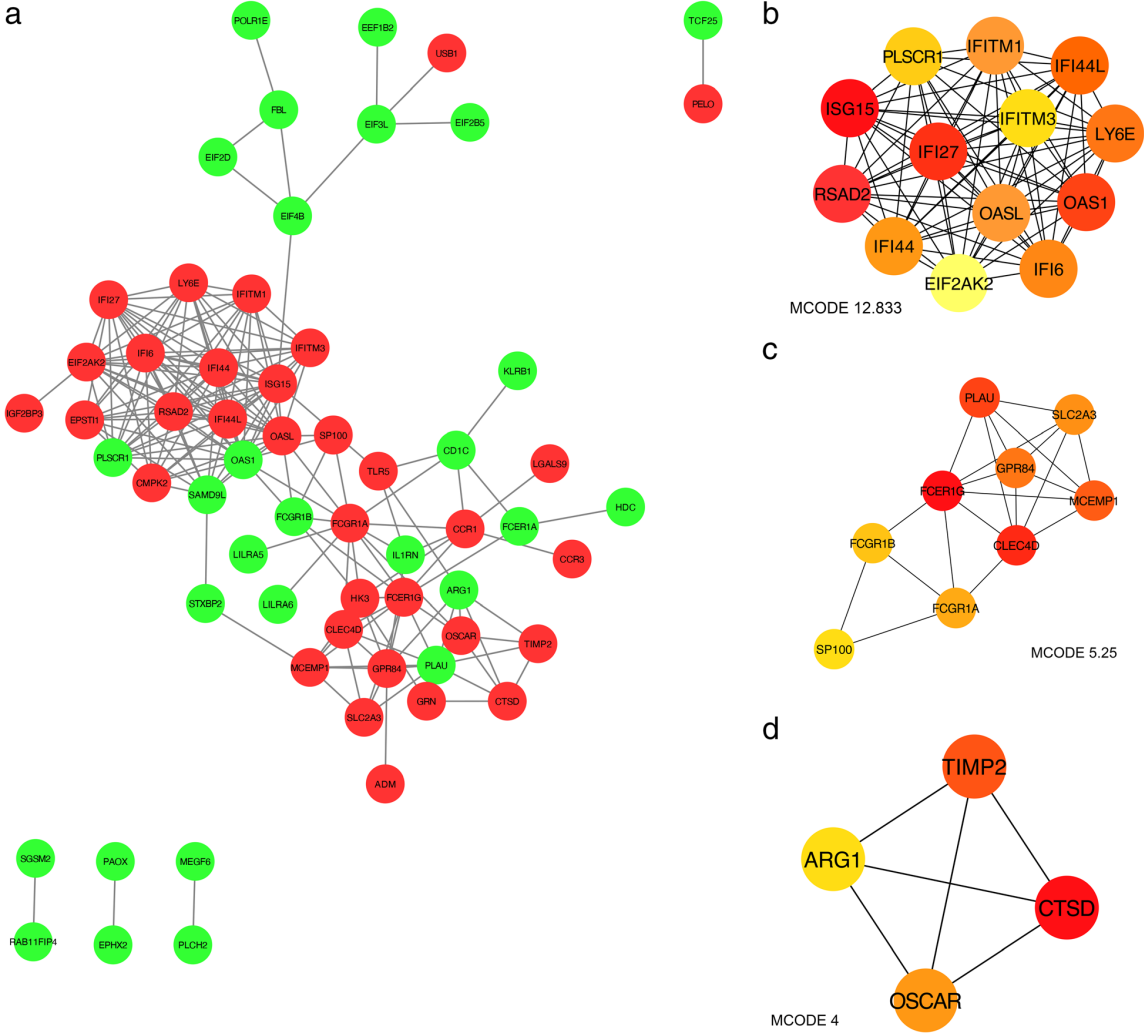
The PPI network of the 131 most significant DEGs. **a** The network contained 61 nodes and 187 edges while DEGs without connection were excluded due to no connection with others. The nodes with red color corresponded to the up-regulated gene while those with green color corresponded to the down-regulated gene. **b**-**d** The significant clusters were extracted from the network. The depth of the color depended on the score calculated by plug-in CytoHubba of Cytoscape. The darker the color, the higher the score was

**Fig. 6:**
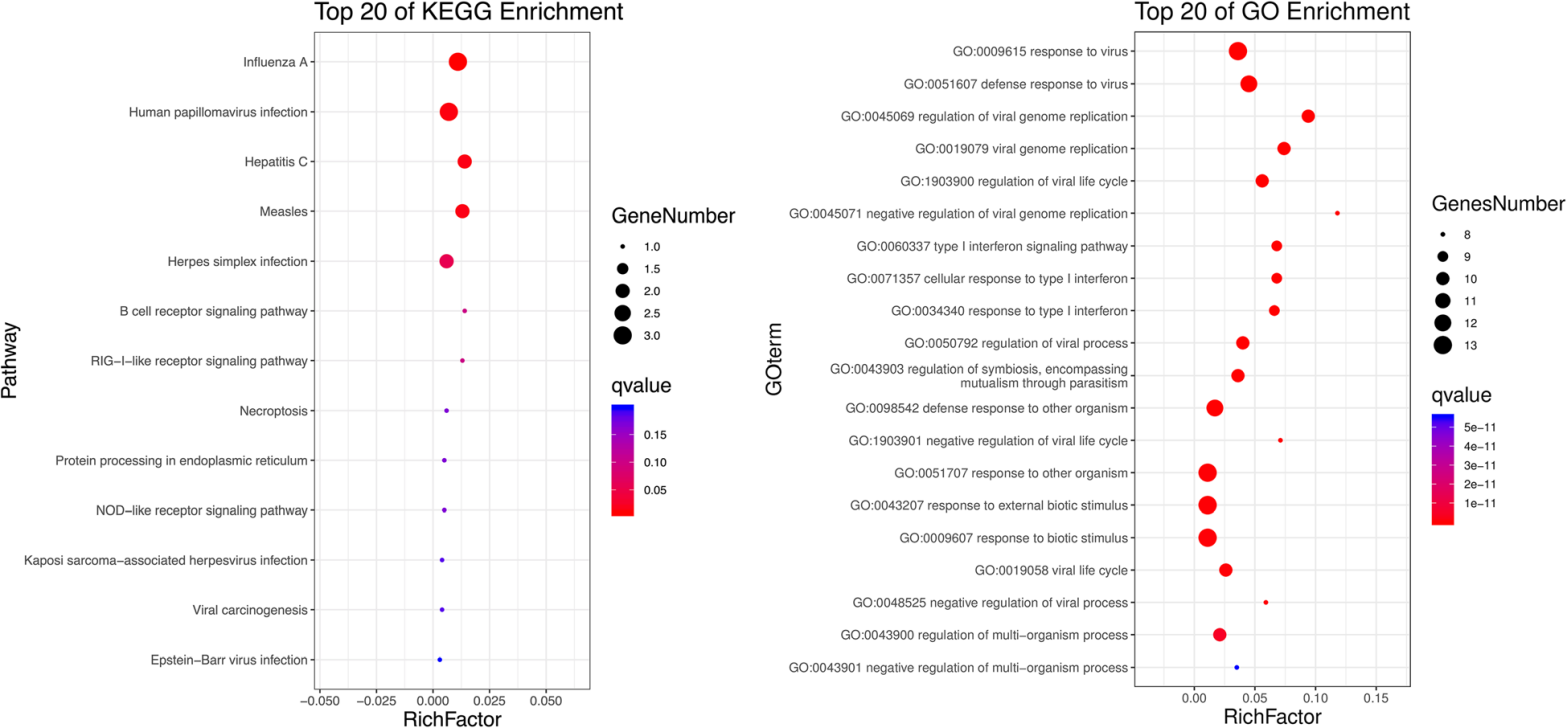
The KEGG and GO enrichment of the thirteen hub genes. The size of dots represented the numbers of genes enriched in each pathway and the color represented the degree of enrichment

The PPI network of the 131 most significant DEGs was constructed (Fig. 5a) containing 61 nodes and 187 edges while DEGs without connection were excluded. Then, the significant clusters were extracted by using plug-in MCODE of Cytoscape software (Fig. 5b-d). The color depth of the nodes represented the different scores calculated by plug-in cytoHubba and the darker of the node, the higher the score was (Fig. 5b-d). The most significant cluster contained 13 nodes and 77 edges and these 13 nodes (IFI27, IFITM1, IFI44, IFI44L, LY6E, OAS1, IFI6, EIF2AK2, RSAD2, ISG15, PLSCR1, OASL and IFITM3) were considered as hub genes (Fig. 5b). The KEGG showed pathways of hub genes were mainly enriched in influenza A, human papillomavirus infection and hepatitis C (Fig. 6a). GO analyses revealed that the hub genes were mainly enriched in response to virus, defense response to virus, regulation of viral genome replication and regulation of viral life cycle (Fig. 6b). The names, abbreviations and functions for these 13 hub genes are shown in Table 1.

**Table 1:** The names, abbreviations and main functions of the 13 hub genes

MCODE score.	Gene symbol	Full name	Function	EPC score
23	IFI27	interferon alpha inducible protein 27	May relate to pathways of Innate Immune System and Interferon gamma signaling.	12.309
14	IFITM1	Interferon Induced Transmembrane Protein 1	May relate to pathways of Class I MHC mediated antigen processing and presentation and B cell receptor signaling pathway	6.575
12	IFI44	Interferon Induced Protein 44	May participate the pathogenesis of Hepatitis D and Limited Scleroderma.	6.469
12	IFI44L	Interferon Induced Protein 44 Like	Diseases associated with IFI44L include Lymph Node Tuberculosis and Aicardi-Goutieres Syndrome.	6.449
12	LY6E	Lymphocyte Antigen 6 Family Member	Diseases associated with LY6E include T-Cell Lymphoma, Subcutaneous Panniculitis-Like and Acute Promyelocytic Leukemia	6.394
11	OASL	2′-5′-Oligoadenylate Synthetase Like	May relate to pathways of Innate Immune System and PI3K-Akt signaling pathway.	6.379
11	IFI6	Interferon Alpha Inducible Protein 6	May play a critical role in the regulation of apoptosis	6.343
11	OAS1	2′-5′-Oligoadenylate Synthetase 1	Activates latent RNase L resulting in viral RNA degradation and the inhibition of viral replication	6.342
11	ISG15	ISG15 Ubiquitin Like Modifie	Chemotactic activity towards neutrophils, direction of ligated target proteins to intermediate filaments, cell-to-cell signaling, and antiviral activity during viral infections	6.319
10	RSAD2	Radical S-Adenosyl Methionine Domain Containing 2	Diseases associated with RSAD2 include Yellow Fever and Chikungunya	6.312
10	EIF2AK2	Eukaryotic Translation Initiation Factor 2 Alpha Kinase 2	Diseases associated with EIF2AK2 include Herpes Simplex and Hepatitis C	6.213
10	PLSCR1	Phospholipid Scramblase 1	May mediate accelerated ATP-independent bidirectional transbilayer migration of phospholipids upon binding calcium ions	6.193
10	IFITM3	Interferon Induced Transmembrane Protein 3	Helps confer immunity to influenza A H1N1 virus, West Nile virus, and dengue virus	6.141

### Expressions of key genes in RSV infection

**Fig. 7:**
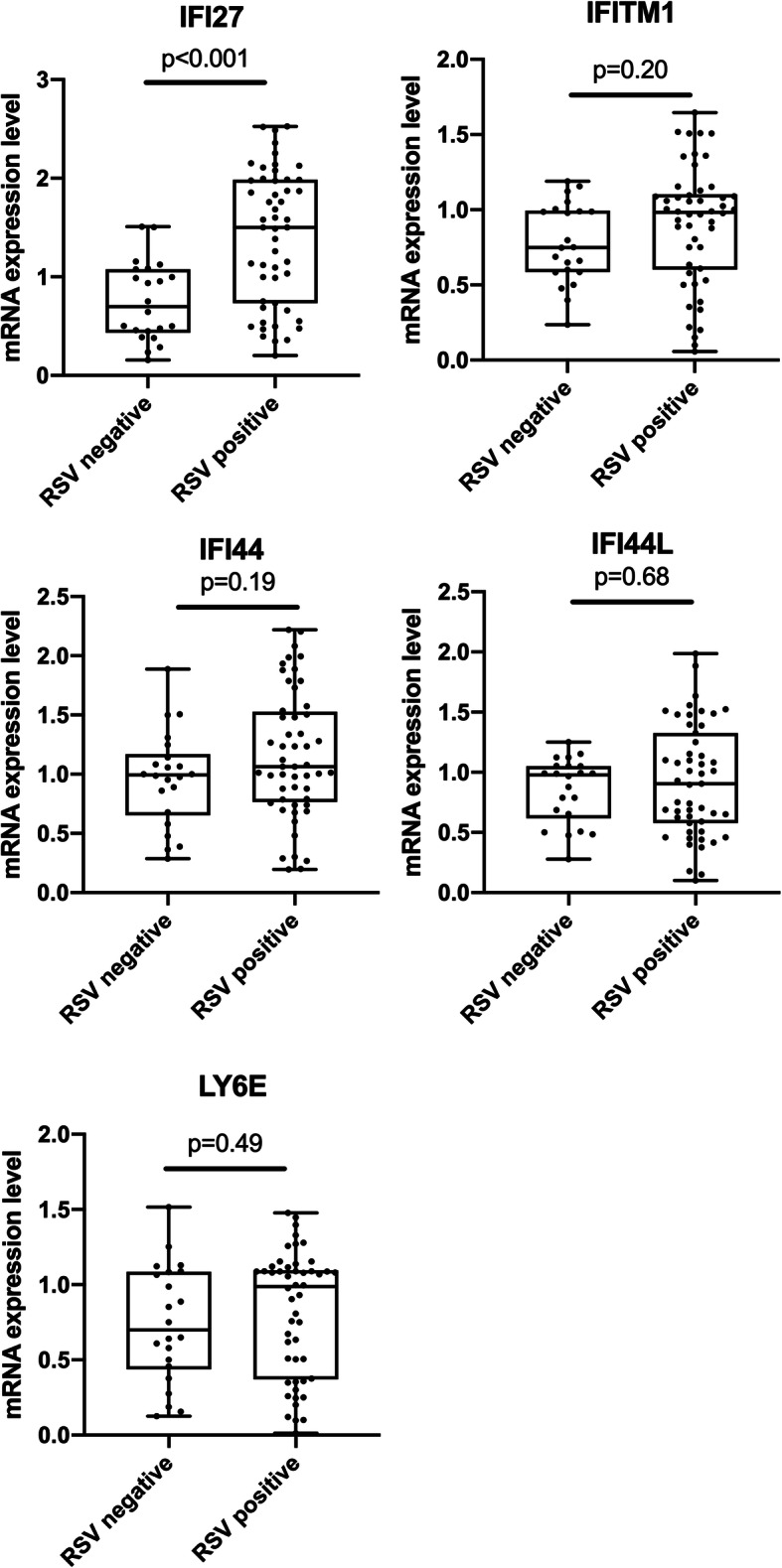
The relative mRNA expression level (2^-ΔCt^) of the top five hub genes (IFI27, IFI44, IFI44L, IFITM1 and LYE6)

The top five hub genes (IFI27, IFITM1, IFI44, IFI44L and LY6E) were further verified by qRT-PCR of clinical samples in our center. IFI27 was much higher expressed in RSV infected patients with a *p*-value< 0.0001 (Fig. 7). While IFITM1, IFI44, IFI44L and LY6E showed no significant difference between RSV infection group and control group (Fig. 7). Then, the 50 RSV infected infants were further divided into two subgroups (low-expression group and high-expression group) by the median mRNA level of IFI27. There was no significant difference in age at first admission due to RSV infection, gender distribution, GA and BW between high-expression IFI27 and low-expression IFI27 groups (Table 2). With respect to body weight at first admission due to RSV infection, patients in high-expression IFI27 group came out with lower body weight comparing with those in low-expression IFI27 group (4.60 ± 1.25 kg versus 6.03 ± 1.84 kg, *p* = 0.018) (Table 2). The high-expression IFI27 was related to significantly more severe cases (13/25, 52.0% versus 6/25, 24%, *p* = 0.041), more requirements of MV (8/25, 32% versus 2/25, 8%, *p* = 0.034), more frequent hospitalization (18/25, 72% versus 5/25, 20% for ≥3 times, *p* < 0.001) and longer cumulative hospital stay (19.0[4.0–41.0] days versus 9.0[5.0–22.0] days, *p* = 0.012) (Table 2).

**Table 2:** The clinical characteristics of the 50 RSV infected patients

Variables	IFI27 expression level		*P*-value
	High-expression (*n* = 25)	Low-expression (*n* = 25)	
Age at first admission (months)^e^	4.7 (1.8–8.7)	5.1 (2.1–11.4)	0.58^a^
Gender, n (%)			
Male	19 (76.0)	17 (68.0)	0.529^c^
Female	6 (24.0)	8 (32.0)	
Body weight at first admission (kg)^e^	4.60 ± 1.25	6.03 ± 1.84	0.018^a^
Gestational age (weeks)	30.8 ± 2.8	32.5 ± 3.5	0.072^b^
Birth weight (kg)	1.34 (0.77–3.25)	1.77 (0.76–3.07)	0.578^a^
Severe cases, n (%)	13 (52.0)	6 (24.0)	0.041^c^
Non-invasive ventilation, n (%)	14 (56.0)	14 (56.0)	0.612^c^
MV needed, n (%)	8 (32.0)	2 (8.0)	0.034^d^
Hospitalization frequency^e^			< 0.001^c^
< 3 times	7 (28.0)	20 (80.0)	
≥ 3 times	18 (72.0)	5 (20.0)	
Cumulative hospital stay (days)^e^	19.0 (4.0–41.0)	9.0 (5.0–22.0)	0.012 ^a^

*RSV* Respiratory syncytial virus, *MV* Mechanical ventilation, ^a^ Mann-Whitney U test; ^b^ Independent-samples T test; ^c^ Pearson Chi square; ^d^ Fisher’s Exact Test; ^e^admission due to RSV infection

### GSEA of IFI27

**Fig. 8:**
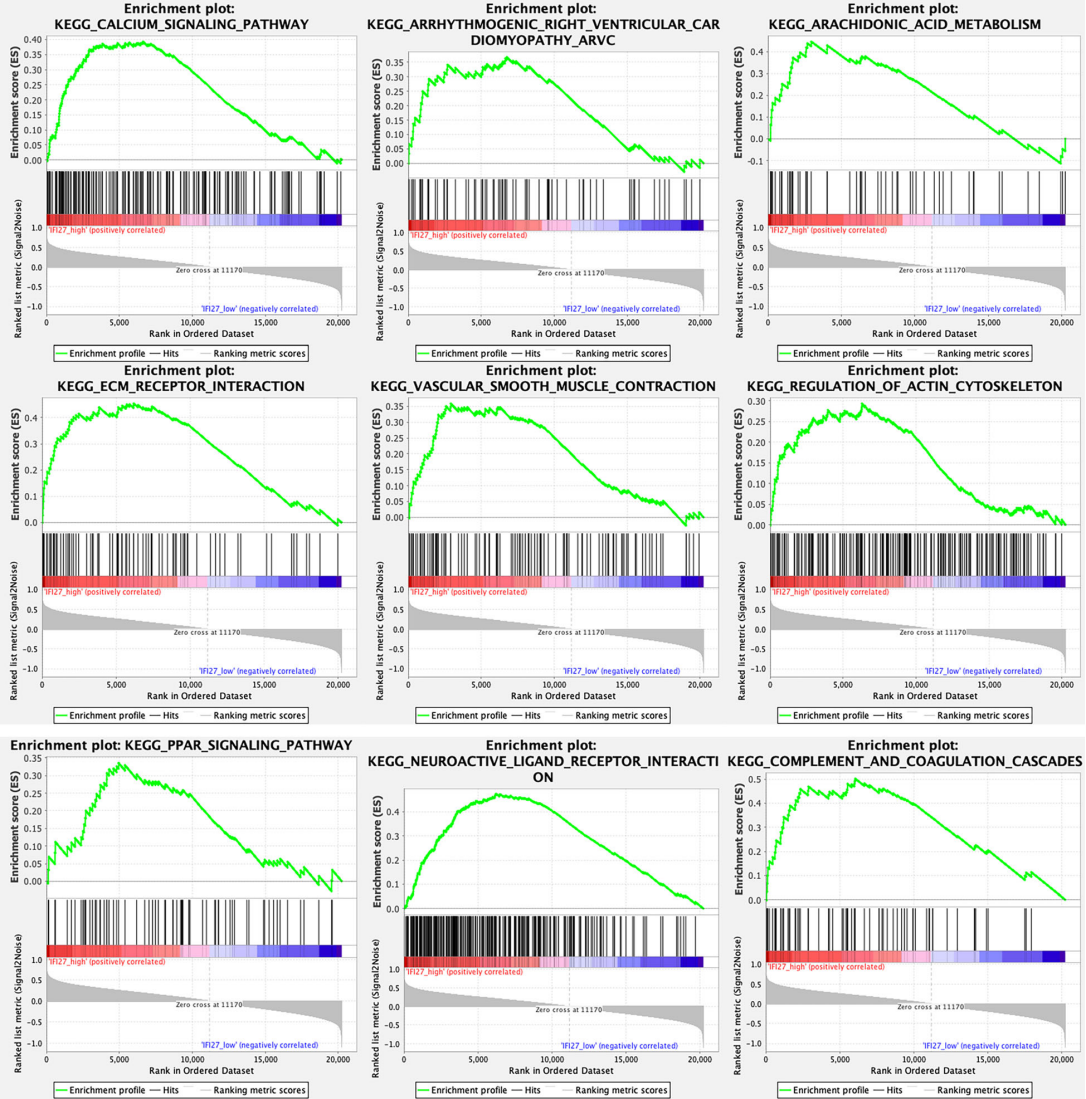
The result of GSEA under the condition of high-expression IFI27

The GSEA revealed that the gene sets associated with calcium signaling pathway, arachidonic acid metabolism, extracellular matrix receptor interaction, vascular smooth muscle contraction, regulation of actin cytoskeleton, PPAR signaling pathway, arrhythmogenic right ventricular cardiomyopathy, neuroactive ligand receptor interaction, as well as complement and coagulation cascades were differentially enriched with the phenotype of high-expression IFI27 (Fig. 8).

## Discussion

RSV infection remains a major cause of LRTIs and hospital visits during infancy and childhood globally. Preterm infants are much more vulnerable to RSV attack and RSV related LRTIs can be much more severe and even fatal for preterm infants [19]. However, the specific mechanism of RSV infection is poorly understood. Therapeutic options on RSV infection are limited to symptomatic treatments and supportive care and show suboptimal efficacy [20]. Recent advances of preventive strategy of immunoprophylactic agent administration (monoclonal antibodies of RSV) shed a light on the prevention of RSV infection [21]. Hence, identifying the candidate genes susceptible to RSV infection could be conducive to early intervention, further diagnose and treatment strategies.

In terms of previous studies, RSV infection among neonates has a strongly genetic background and a few independent microarray platforms have performed the DGEs analysis comparing RSV infected patients with healthy people [22]. However, attributing to the heterogeneity between different platforms, the results yielded from one single independent platform may not be reliable. Thus, it is reasonable and meaningful to screen and identify the candidate genes from multiple microarray datasets and platforms. In the present study, to minimize the false positive rates generated from multiple microarray platforms, three datasets were normalized and merged as one expression matrix. The study batch effect was adjusted and the DEGs were calculated by meta-analysis. Totally, 4028 DEGs were identified and among which, 131 most significant DEGs were selected. To further select the hub nodes and understand the functions and pathways of the 131 most significant DEGs, the PPI network was constructed and KEGG and GO enrichments were also performed subsequently. Accordingly, thirteen DEGs were screened out as hub genes and were sorted to be involved in response to virus, defense response to virus, regulation of viral genome replication and regulation of viral life cycle. Besides, IFI27 showed the highest EPC score of 12.309 and was confirmed to be the most significant expressed either in bioinformatical analysis or in clinical sample verification.

IFI27 is a member of the interferon alpha inducible proteins that may participate in the pathogenesis of various viral infections [23]. IFI27 was confirmed to be involved in a series of biological pathways: innate immune, interferon gamma signaling, RNA polymerase II activating transcription factor binding and lamin binding [24]. Tang et al [25] showed that IFI27 alone provides equivalent diagnostic capability comparable to that of multi-gene biomarkers in differentiating between influenza and bacterial infections. In addition, IFI27 also plays a proinflammatory role by inducing the nuclear export of an anti-inflammatory nuclear receptor, NR4A1 [26]. Consistently, our pathway enrichment analysis showed that IFI27 was involved in the immune system and response to virus.

As an interferon-α inducible protein, IFI27 was confirmed to be up-regulated in PBMCs of systemic lupus erythematosus patients [27] and in the lungs after influenza A infection in mice, mainly due to the infiltration of macrophages and lymphocytes [28]. It is also up-regulated in inflammatory psoriatic skin and in some epithelial cancers, such as ovarian cancer, and its expression is associated with patient survival [29]. IFI27 is a novel modulator of innate immune response and it regulates anti-inflammatory nuclear receptors. Experiments in IFI27-deficient mice indicated that a lack of IFI27 prolongs survival in experimental sepsis and endotoxemia [30]. These observations suggest that IFI27 is involved in the regulation of inflammatory events in PBMCs. It is confirmed that immunological reaction gets involved in viral infectious diseases and such immunological reaction is not only limited in connective tissue diseases and cancers. Therefore, as a highly immune-connected gene, we can rationally deduce that IFI27 may also has a strong correlation with severe RSV disease. A latest research published in 2020 conducted by Min Zhu et al. revealed that IFI27 may be served as a potential indicator for severe enterovirus-71 caused hand foot and mouth disease [31].

Altogether, IFI27 may be associated with RSV progression by multiple functions in activating immune system, regulation of viral genome and proinflammation. Thus, IFI27 may be served as a gene signature for diagnosis and therapeutic target of RSV infection. In further clinical verification, it was demonstrated that infants with relatively high IFI27 expression suffered from more incidence of severe cases, more requirements of invasive ventilation, more frequent hospitalization as well as longer hospital stay. Therefore, IFI27 may also get involved in the progression of RSV infection and may serve to predict the severity of RSV infection. By now, many researches about IFI27 focus on the connective tissue diseases and tumors whereas the studies regarding the correlation between IFI27 expression and viral infectious diseases are inadequate, therefore, it is meaningful and worthful to pay more attention on this topic.

Another four hub genes selected by bioinformatical analysis were IFITM1, IFI44, IFI44L and LY6E, respectively. Interferon induced transmembrane protein 1 (IFITM1) is a member of interferon-induced transmembrane proteins and can be activated by various viruses. IFITM1 inhibits the entry of viruses to the host cell cytoplasm, permits endocytosis and prevents subsequent viral fusion and release of viral contents into the cytosol [32–34]. Interferon induced protein 44 (IFI44) and interferon induced protein 44 like (IFI44L), an important paralog of IFI44, are associated with the formation of microtubular structures as well as exhibiting a low antiviral activity against hepatitis C virus [33, 35, 36]. Lymphocyte antigen 6 family member E (LY6E) may participate in T-cell development, metabolism of proteins and ectoderm differentiation [37–39]. However, their significance in RSV infection is ambiguous since the clinical samples in our center showed no significant difference between groups.

Several limitations about our present study should be considered. Firstly, we did not compare RSV infected patients with patients whose respiratory tract infection were caused by non-RSV viruses. Actually, as a member of the interferon alpha inducible proteins, IFI27 may participate in the pathogenesis of various viral infections not solely limited in RSV infection. Even so, during infancy, RSV still remains the biggest threaten to preterm infants and early recognition of the status of IFI27 expression could provide certain values in clinical decision-making and disease prevention. Secondly, the clinical samples (*n* = 72) were not adequate enough, which may lead to considerable bias of the result. We hope we could expand our sample size in the oncoming research.

In summary, our study may provide a novel understanding of the mechanism of RSV infection. Several key genes were screened out and were further verified by clinical samples. IFI27 was confirmed to be the most meaningful one that highly up-regulated in RSV infected infants. IFI27 may have the potential for screening preterm infants who are susceptible to RSV infection and for predicting the severity of RSV infection. In-depth researches of those genes identified in present study need to be further explored.

## Conclusions

Our study established a gene signature involved in pathogenesis of RSV infection and demonstrated that IFI27 might be an optimal biomarker for predicting and evaluating the severity of RSV infection in preterm infants.

## Supplementary Information

**Additional file 1: Table S1.** The general features of the 72 infants.

**Additional file 2 Table S2.** The sequences of the top five hub gene primers.

**Additional file 3: Figure S1.** The principal component analysis (PCA) of the three datasets before and after the study batch effect adjusted. Blue dots represented RSV infected patients while red dots represented healthy controls. The shape of dots represented which dataset they were from.

## Data Availability

The part of original data for bioinformatical analysis can be found at GEO database (http://www.ncbi.nlm.nih.gov/geo) and the part of the clinical data could be obtained from the authors if the editor or reviewer request.

## Abbreviations

BP: Biological process
BW: Birth weight
CC: Cell component
DEG: Differentially expressed gene
DAVID: Database for annotation, visualization and integrated discovery
FC: Fold-change
FDR: False discovery rate
GA: Gestational age
GEO: Gene expression omnibus
GO: Gene ontology
GSEA: Gene set enrichment analysis
IFI27: Interferon alpha inducible protein 27
IFI44: Interferon induced protein 44
IFI44L: Interferon induced protein 44 like
IFITM1: Interferon induced transmembrane protein 1
KEGG: Kyoto Encyclopedia of Genes and Genomes
LRTI: Lower respiratory tract infection
LY6E: Lymphocyte antigen 6 family member E
MCODE: Molecular Complex Detection
MF: Molecular function
MV: Mechanical ventilation
PBMC: Peripheral blood mononuclear cell
PPI: Protein-protein interaction
qRT-PCR: Quantitative real-time polymerase chain reaction
RSV: Respiratory syncytial virus
STRING: Search Tool for the Retrieval of Interacting Genes
