# Supplementary material for: IFI27 may predict and evaluate the severity of respiratory syncytial virus infection in preterm infants
Source: Hereditas. 2021 Jan 2;158:3. doi: 10.1186/s41065-020-00167-5 (PMC7778825; doi:10.1186/s41065-020-00167-5)
Supplement: Supplementary file 1 — Additional file 1: Table S1. The general features of the 72 infants. [file 41065_2020_167_MOESM1_ESM.docx]

Supplementary Table 1 The general features of the 72 infants

| Variables | RSV-positive (n=50) | RSV-negative (n=22) | P-value |
| --- | --- | --- | --- |
| Gestational age (weeks) | 31.6±3.2 | 32.7±4.1 | 0.12^a^ |
| Birth weight (kg) | 1.56 (0.76-3.25) | 1.90 (0.79-4.02) | 0.24^b^ |
| Gender, n (%) |  |  |  |
| Male | 36(72.0) | 11(50.0) | 0.07^c^ |
| Female | 14 (28.0) | 11(50.0) |  |

RSV: respiratory syncytial virus; ^a^ Independent-samples T test; ^b^ Mann-Whitney U test; ^c^ Pearson Chi square
