# Supplementary material for: IFI27 may predict and evaluate the severity of respiratory syncytial virus infection in preterm infants
Source: Hereditas. 2021 Jan 2;158:3. doi: 10.1186/s41065-020-00167-5 (PMC7778825; doi:10.1186/s41065-020-00167-5)
Supplement: Supplementary file 2 — Additional file 2 Table S2. The sequences of the top five hub gene primers. [file 41065_2020_167_MOESM2_ESM.docx]

Supplementary Table 2 The sequences of the top five hub gene primers

| IFI27 | Forward | 5'-TGCTCTCACCTCATCAGCAGT-3' |
| --- | --- | --- |
|  | Reverse | 5'-CACAACTCCTCCAATCACAACT-3' |
| IFITM1 | Forward | 5'-CCAAGGTCCACCGTGATTAAC-3' |
|  | Reverse | 5'-ACCAGTTCAAGAAGAGGGTGTT-3' |
| IFI44 | Forward | 5'-ATGGCAGTGACAACTCGTTTG-3' |
|  | Reverse | 5'-TCCTGGTAACTCTCTTCTGCATA-3' |
| IFI44L | Forward | 5'-AGCCGTCAGGGATGTACTATAAC-3' |
|  | Reverse | 5'-AGGGAATCATTTGGCTCTGTAGA-3' |
| LY6E | Forward | 5'-CAGCTCGCTGATGTGCTTCT-3' |
|  | Reverse | 5'-CAGACACAGTCACGCAGTAGT-3' |
| GAPDH | Forward | 5'-CCAGGTGGTCTCCTCTGA-3' |
|  | Reverse | 5'-GCTGTAGCCAAATCGTTGT-3' |
