# Supplementary figures and images for: IFI27 may predict and evaluate the severity of respiratory syncytial virus infection in preterm infants
Source: Hereditas. 2021 Jan 2;158:3. doi: 10.1186/s41065-020-00167-5 (PMC7778825; doi:10.1186/s41065-020-00167-5)

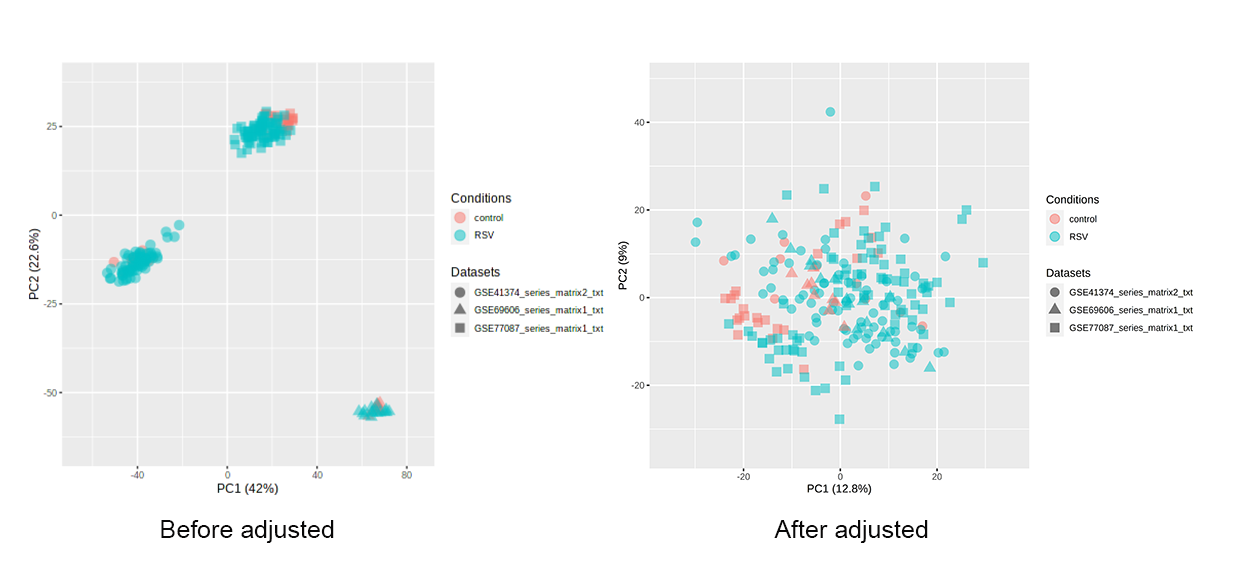

Supplement: Supplementary file 3 — Additional file 3: Figure S1. The principal component analysis (PCA) of the three datasets before and after the study batch effect adjusted. Blue dots represented RSV infected patients while red dots represented healthy controls. The shape of dots represented which dataset they were from. [file 41065_2020_167_MOESM3_ESM.tif]
